# Influence of Groundwater pH on Water Absorption and Waterproofness of Polymer Modified Bituminous Thick Coatings

**DOI:** 10.3390/ma14092272

**Published:** 2021-04-27

**Authors:** Barbara Francke, Maria Wichowska

**Affiliations:** Building Research Institute, Filtrowa 1, 00-611 Warsaw, Poland; m.wichowska@itb.pl

**Keywords:** polymer modified bituminous thick waterproofing coatings, water absorption, durability of waterproofing

## Abstract

Polymer modified bituminous thick coatings are increasingly used in the construction industry to protect underground parts of buildings from groundwater. When assessing their durability, one vital issue related to their functional properties is the influence of water absorption on the waterproofness of the applied solution as a result of the action of groundwater with different pH values. As part of the research, the water absorption of the products in question was assessed using the method of total immersion in water with pH of 4.0, 7.0 and 7.5 as well as comparatively, as a result of one-way exposure to demineralized water under successively increasing pressure up to 0.5 MPa. The moisture susceptibility of the coatings was assessed both concerning the local surface damage and the continuous waterproofing coating. It was established that the coatings show the highest water absorption when the water pH is 4.0, which simulates the groundwater aggressiveness on construction products. It was proven that moisture absorbed by the coatings is retained within this layer and is not transferred to the substrate on which the coatings are laid. It was also found that water in contact with the tested coatings changes its reaction to alkaline, which can result in contamination of groundwater in the area of waterproofing coating. A modification of the method of assessing the water absorption of polymer modified bituminous thick coatings was proposed, taking into account their use in conditions of use.

## 1. Introduction

The majority of processes which destroy building materials take place in the presence of water or moisture and that is why structures must be protected from the ingress of unwanted rainwater, water accumulated in the soil [1]. The application of such protection makes it possible to guarantee the comfort of using indoor spaces, which in the case of buildings indirectly affects the health and lives of their inhabitants. Each building should be protected against the ingress of unwanted rainwater or water accumulated in the ground or on the surface of terraces and balconies, as well as water splashed on the floor of “wet” rooms and delivered there from the plumbing system. Underground portions of buildings are subject to constant exposure to water and moisture stored in the surrounding soil and to ground pressure. The total pressure varies from 30 to 60 MPa for each 0.3 m depth [2]. Of course, these values are lower for dry and permeable soils, and increased for cohesive soils. Waterproofing of below ground structures of buildings should be a continuous and tight system separating buildings or their parts from water or water vapour. For such installations, both plastic and rubber damp proof and basement tanking sheets, bituminous membranes and coatings are used [3,4], among others polymer modified bituminous thick coatings of a thickness exceeding 3 mm but usually no thicker than 5 mm [5,6,7]. The functional properties of polymer modified bituminous thick coatings for waterproofing are stated in EN 15814 [6] standard. Bituminous thick coatings are usually applied manually by spackling, but spray application is also possible. The completed waterproofing coatings should adhere well to the substrate and, at the same time, be highly waterproof and of an adequate thickness to provide protection against water in ground-water conditions. Bituminous thick coatings were first introduced in Germany [7,8,9,10,11]. They started to be applied in Europe fairly quickly. In many countries, to describe these materials, the KMB (German for kunststoffmodifizierte bitumendickbeschichtungen) is used. Coatings made from these components have several properties unusual for waterproofing products. The first of these is significant water absorption, sometimes reaching up to several weight percent. The need to assess this property was not included in EN 15814 [6] standard. Bituminous thick coatings are one or two components pastes suitable for filling or spraying. Whether a 1-component or 2-component material is used, it is a factory-prepared mixture consisting of bitumen in the form of anionic or cationic asphalt emulsion, plastics and fillers. The mixture might also include added fibre. Fillers can include, e.g., polystyrene granules, rubber granules or mineral fillers. The second component in 2-component products is usually a powder–it contains cement, e.g., aluminous cement, and powdered highly hygroscopic substances. Hardening occurs as a result of drying–the evaporation of water; in 2-component products, drying is accelerated by the binding of excess water by a second component which, however, should not be regarded as a chemical hardener. As asphalt emulsions are the main constituent of these products, a brief summary of the effects of different modifications of the asphalt used to produce asphalt emulsions on the properties of the mixture obtained is given below. The modifiers and additives which have been used to boost bitumen performance include polymers, chemical modifiers, extenders, oxidants and antioxidants, hydrocarbons, and anti-stripping additives [12]. Polymer-modified bitumens (PmBs) are produced by the mechanical mixing or chemical reactions of a bitumen and one or more polymer in a percentage usually ranging from 3% to 10%, relatively to the weight of bitumen. In the first case, no chemical reactions occur between the two partners in the system and polimer is considered as a filler which gives specific properties to the mixture. In the second case, the mixtures are said to be complex, because chemical reactions or some other interaction occurs between the two partners in the system [13]. Modified bitumens are characterized as a two-phase system: bituminous, prevalently as asphaltenic matrix, and polymeric matrix. From a bitumen/polymer interaction mechanism point of view, according to Polacco et al. [14], polymer modification results in a thermodynamically unstable but kinetically stable system in which the polymer is partially swollen by the light bitumen components (maltenes) and can swell up to nine times of its initial volume [15]. Polymers tend to induce the micelles aggregation of the asphaltenes or to increase their degree of association, according to the nature of the original bitumen. Therefore, associated asphaltene micelles can settle to the bottom of the blend during static hot storage. According to this mechanism, the degree of phase separation of polymer modified binders can be influenced by storage conditions such as temperature and time. As shown by Lu et al., the phase separation will mainly be governed by the nature of the base bitumen and the characteristics and content of the polymer [16]. To date, different types of additives and polymers have been used for bitumen modification [17]. The elastomer rubber was the earliest asphalt modifier, and it offers advantages in terms of low-temperature cracking resistance, elastic properties, and toughness [18,19,20]. Combining the properties of both rubber and polymer resins, styrene butadiene styrene (SBS) can comprehensively improve the properties of base asphalt and, currently, become the most used, and studied, asphalt modifier [21,22,23,24]. Additives help to improve the performance of asphalt, they also create some problems, such as the compatibility of the modifier and the base asphalt [25], stability of the modified-asphalt [26], and the balance between high- and low-temperature properties [27]. To solve these problems, many researchers added nanomaterials into base asphalt or polymer-modified asphalt (PMA) to make up for the deficiencies of PMA in performance. Polacco et al. [28], Zhang et al. [29,30] and other scholars believe that the exfoliated and intercalated structure is formed in a nano-layered material/polymer/asphalt system. Which can separate oxygen and prevent the volatilization of the light-asphalt components, thereby, increasing the aging resistance of asphalt and improving the service life of a modified asphalt binder.

Unfortunately, available literature refers to bitumen used in the production of road surfaces or bitumen used in the production of membrane coating compounds. There are no literature reports on the resistance of bituminous waterproofing coatings to long-term exposure to water. The authors have conducted research [7] in order to assess the influence of the aforementioned water absorption by the described coatings on their durability in the Polish weather conditions, attempting to determine whether the moisture is retained within the structure of the product or is transferred to the substrate. The other of the properties is a significant deformation susceptibility under repeated operational load, which in extreme cases may lead to layer damage. Due to this reason, coatings made of these materials, before backfilling with soil, require additional surface protection. An additional problem discussed in publications is the fact that the above-mentioned coatings are leaching by groundwaters, which contributes to their contamination [31]. One of the basic components of the above-mentioned coatings is bitumen which is a mixture of high molecular weight organic compounds, mostly hydrocarbons with carbon numbers greater than C25. Contains small amounts of polycyclic aromatic hydrocarbons (PAH) and various trace metals [32]. Although most of the PAH is not bioavailable Fagbote and Olanipekun [33] and Olajire et al. [34] reported an increase of PAH in surface waters and sediments samples from the bitumen belt in Nigeria. According to Olajire et al. [34] the total PAH concentrations of water samples were high enough to cause acute toxicity to exposed organisms. Kelly et al. [35] examined the concentrations of Sb, As, Be, Cd, Cr, Cu, Pb, Hg, Ni, Se, Ag, Tl, and Zn in surface waters and melted snow in Alberta. They found an increase resulting from upgrading facilities of the oil sand. Olabemiwo et al. [36] tested the impacts of bitumen leachate on rats. The leachate contained sulphate, nitrate, hydrocarbons and heavy metals. It turned out that the leachate had a ‘‘very negative implication on the health status of the rat’’. These results give only hints concerning the environmental compatibility of bitumen waterproofing, because the leaching conditions are quite different. Waterproofings have a closed surface. They are not percolated or even stirred with water [31] like the leachate examined by Olabemiwo et al. [36]. Nevertheless, the leaching of the mentioned pollutants should be considered when using bitumen in contact with groundwater.

The aim of the research discussed in this paper was to determine how the water absorption of polymer modified bituminous thick coatings changes concerning different water pH and the trend of these changes in different operating conditions. The assumption concerning the need for assessment of the influence of water of different pH on the water absorption of the discussed coatings resulted from the earlier experience of the authors [7] indicating the difference in the results obtained in this scope, related to the chemical contamination of water in the soil environment. An additional element of the assessment was an attempt to determine whether groundwater with different pH undergoes deamination—a change in pH during contact with waterproofing coatings made of polymer modified bituminous thick coatings on ground walls. For this reason, after removing the samples from a liquid medium, the pH of the solutions in which they were soaked was determined. In the tests, water with three different pH values was used, starting from demineralized water with a pH of 7, traditionally used to assess the absorption of products in laboratory conditions, through typical tap water with a pH of 7.5, ending with water with a pH of 4, which according to EN 206 [37] is accepted for the assessment of construction substance exposed to groundwater aggressiveness. In the case of water with a pH of 4, two test variants were assessed, i.e., samples without the additional protection of the cut edges of the samples, which reflects the condition of possible mechanical damage of the coatings in question, which may occur during operation, especially in the case of soft coatings such as polymer modified bituminous thick coatings. The second case is that of samples with cutting edges additionally protected by a layer of wax, which was performed in order to reflect the behaviour of coatings concerning which no additional mechanical surface damage is found. The next stage of the research was to determine the influence of water absorption of polymer modified bituminous thick coatings on solutions applied concerning waterproofing, especially with high-pressure water impact on foundations. The results obtained proved that moisture absorbed by the tested waterproofing coatings at periodically increasing water pressure remains enclosed within the coating structure and is not transferred to the substrate applying periodical water pressure up to 0.5 MPa. In the tests concerning waterproofness, demineralized water with a pH of 7.0 was also used, which made it possible to compare the water absorption of polymer modified bituminous thick coatings as a result of long-term, one-sided application of water to the coating at changing water pressure values with the water absorption of the same coatings determined as a result of total immersion for 24 h, with the water on all sides of the samples. To determine the identification properties of polymer modified bituminous thick coatings, the authors’ own method of water absorption assessment was adopted. In addition, it was found that the leachates formed during total immersion of polymer modified bituminous thick coatings samples in an aqueous solution with different initial pH values, i.e., 4.0, 7.0 and 7.5 change significantly towards alkaline, which may indicate the leaching of coatings contributing to the contamination of groundwater.

## 2. Materials and Methods

### 2.1. Materials

Since the components of polymer modified bituminous thick coatings are manufactured in 1- and 2-component versions and the liquid component may contain both polystyrene filler with grain size up to 1 mm and fine-grained mineral filler, the choice of materials for testing was guided by the need to compare the properties of the products contained in all of the above-mentioned assortment groups. After the preliminary study, four representative products were selected for further testing, i.e., three 2-component and one 1-component products. In both groups, one representative sample was selected for products with polystyrene filler with grain size of 1 mm, which is often used in both 2-component and 1-component products. The other two samples were 2-component products with a traditional mineral filler. Since products with such a filler constitute the largest percentage in the group of polymer modified bituminous thick coatings, two products from this group, produced by two different manufacturers, were selected for testing (i.e., samples 3 and 4). The properties of the tested products are:

- Sample 1—two-component, solvent-free sealing compound based on asphalt, plastics and fillers, with a polystyrene filler, non-volatile components—67%, water in a liquid component—30%, density in a mineral component—(1.10–1.35) g/cm^3^, bulk density in a liquid component—(0.6–0.75)g/cm^3^, waterproofness Class W2B (at a pressure of 0.075 N/mm^2^ for 72 h), no sliding from vertical surface at 70 °C for 2 h.
- Sample 2—one-component, solvent-free waterproofing coating with polystyrene filler, mineral content—19.6%, water content—30%, waterproofness Class W2A (at a pressure of 0.075 N/mm^2^ for 72 h—with the reinforcement mesh), crack bridging ability Class CB2 (no damage at ≥2 mm wide crack), and compressive strength Class C2A (0.30 MN/m^2^—with reinforcement mesh),
- Sample 3—two-component, solvent-free waterproofing coating, waterproofness Class W2A (at a pressure of 0.075 N/mm^2^ for 72 h—with the reinforcement mesh), crack bridging ability Class CB2 (no damage at ≥2 mm wide crack) and compressive strength Class C2A (0.30 MN/m^2^—with reinforcement mesh),
- Sample 4—two-component, solvent-free waterproofing coating, non-emulsified asphalt—1.47%, water in the liquid component—37%, waterproofness Class W2A (at a pressure of 0.075 N/mm^2^ for 72 h—with the reinforcement mesh), crack bridging ability Class CB2 (no damage at ≥2 mm wide crack), and compressive strength Class C2A (0.30 MN/m^2^—with reinforcement mesh).

A supplementary list of basic functional properties of tested products is provided in Table 1.

The coatings were made according to the manufacturers’ instructions, two-component products were mixed in the proportions given in Table 1. The first two samples, with polystyrene filler, are thicker than samples 3 and 4, which is due to the mineral filler. All samples comprised of coatings without additional reinforcement using reinforcement mesh.

### 2.2. Methods of Tests

#### 2.2.1. Water Absorption of the Coating

The study of polymer modified bituminous thick coatings water absorption was designed to determine how the water absorption changes for different values of pH and the trend of these changes in different operating conditions. Test samples were cut from coatings made from specimens 1 through 4 with the properties listed in Section 2.1. Coatings were made on silicone-treated paper, according to the manufacturer’s instructions. and then seasoned for 28 days at (23 ± 2) °C and (50 ± 5)% RH (relative humidity). After completing seasoning process, the coatings were removed from the substrate and five square samples, each measuring 50 mm ± 1 mm on a side and as thick as the product was cut into four batches of samples from each coating to be tested. Only in one series of samples were the cut edges of the squares protected by liquid wax, while in the remaining 3 series the edges of the samples were left without additional surface protection. The samples prepared in this way were seasoned at (23 ± 2) °C and (50 ± 5)% RH for at least 48 h and then weighed with an accuracy of 0.001 g and placed in 16 water baths. Next, the samples were totally immersed for (24 ± 1) hours in water at (23 ± 2) °C, respectively: series 1—in demineralized water with a pH of 7.0, series 2—in tap water with a pH of 7.5, series 3—in water with a pH of 4 (samples without protected cut edges), series 4—in water with a pH of 4 (samples with cut edges protected with wax). HNO_3_ acid was used to create a solution with a pH of 4. In all cases, the pH of the solution was determined using potentiometric measurement according to EN 12850 [38]. The samples were immersed in containers with covers holding min. 2.5 L of the test solution, allowing to place five 50 mm × 50 mm samples. During the test, the samples were immersed in water and did not come into contact with each other or the walls of the container. Next, the samples were removed from the water, dried on both sides with filter paper and weighed within max 1 min with an accuracy of 0.001 g.

Water absorption was calculated as a percentage according to the Equation (1):

X = ((m_1_ − m_o_)/m_o_) × 100% (1)

where:

X—water absorption, %,

m_o_—sample weight after conditioning, g,

m_1_—sample weight after immersion, g.

The result was the arithmetic mean of the 5 tests.

#### 2.2.2. Waterproofness Test

**Figure 1:**
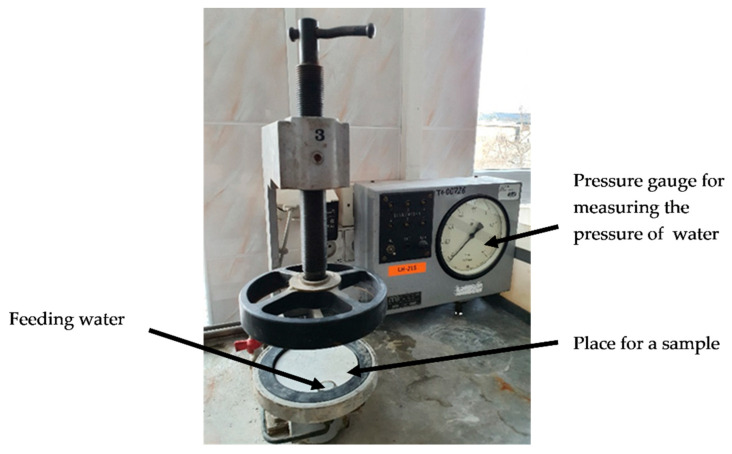
Equipment for testing water absorption of coatings.

The assessment of waterproofness of samples 1–4 was performed on samples of coatings applied directly on concrete substrates, in the shape of disks, each 15 cm in diameter, 3 cm thick, made of permeable concrete, i.e., leaking under a pressure of 0.15 MPa within 3–5 h. The aim of the test was to determine whether moisture absorbed during the test by the waterproofing coating, at cyclically increasing pressure is retained within the coating layer or is transferred to the substrate surface. In the tests, demineralized water with a pH of 7.0 was used, which made it possible to compare the water absorption of polymer modified bituminous thick coatings as a result of long-term, one-sided application of water to the coating at changing water pressure values with the water absorption of the same coatings determined as a result of total immersion for 24 h, with the water on all sides of the samples. Coatings were applied directly to the aforementioned concrete substrates which were primed with bitumen emulsion recommended by the manufacturer and seasoned for 28 days at (23 ± 2) °C and (50 ± 5)% RH. Next, the samples were placed in the test equipment, shown in Figure 1, which allows applying water pressure on the application side of the coating.

The test consisted in exposing the samples to demineralized water at a pressure of 0.15 MPa for 7 days, and if there was no water leakage after this time, the pressure was increased to 0.2 MPa and then by a further 0.1 MPa every 24 h until the pressure causing the leakage was reached. The result was considered positive if all three samples tested showed no leakage at a given pressure value. In addition, the concrete substrates were weighed to the nearest 0.01 g prior to application of the coatings. This was repeated for test substrates with seasoned coating layers as well as carried out after the test. On the basis of the obtained results, the values of water absorption of the samples after pressurized water treatment were also determined. After taking all measurements, the coatings were removed from the concrete substrate and the concrete substrate was additionally broken up, checking visually, whether or not the substrate was wet inside.

## 3. Results

Table 2, Table 3 and Table 4 show the results of water absorption test for samples 1–4 immersed for 24 h in demineralized water at pH 7.0 (Table 2), tap water (Table 3) and water at pH 4 (Table 4).

**Figure 2:**
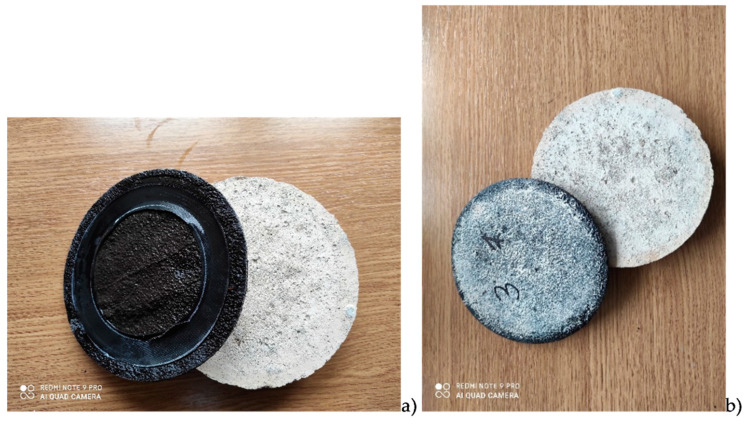
Samples after the waterproofness tests: (**a**) top side of the sample after removal from the concrete substrate, (**b**) bottom side of the sample. Next to it, there is a concrete substrate.

Table 5 shows the results of the waterproofness test of coatings subjected to increasing water pressure, supplemented with the values of the water absorption of the coatings after the waterproofness test. Figure 2 shows the coating samples after removal from the concrete substrate after the test.

## 4. Discussion

**Figure 3:**
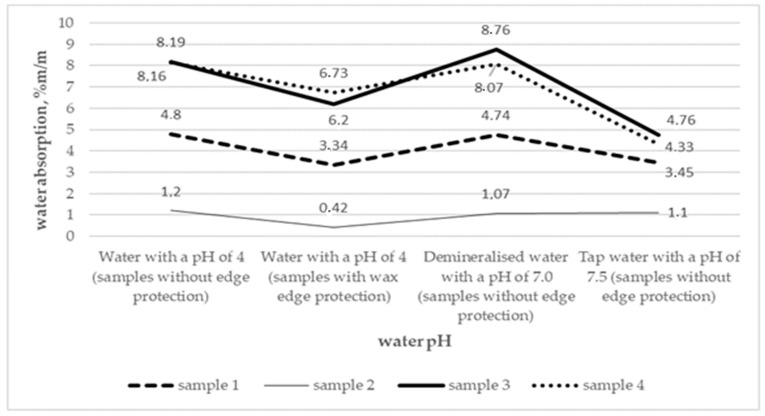
Trend lines concerning the changes in water absorption of samples 1–4 as a function of the pH value of the test solution and the method of protecting the edges of the samples.

Figure 3 graphically presents a comparison of the water absorption values of tested coatings concerning different test conditions and the trend lines of changes in the water absorption of individual coatings depending on the pH of the test solution.

As the comparison suggests, demineralized water and water with a pH of 4 (for samples without the additional protection of edges) contribute to the highest increase in water absorption of the three 2-component polymer modified bituminous thick coatings and these values are significantly higher than those obtained when the coatings are immersed in tap water. Only in the case of the one-component coating (sample 2) both values are at a similar level. In this case, the structure of the cut edges of the samples without additional protection has no influence on the increase in water absorption of the coating after 24 h of exposure to both demineralized water with a pH of 7.0, tap water with a pH of 7.5 and water with a pH of 4, obtaining values of 1.07%, 1.1% and 1.2% respectively. Significantly lower water absorption values for the one-component coating (sample 2) were obtained after the samples with cut wax-protected edges were soaked in water with a pH of 4, i.e., 0.42%. In this case, it is evident that the open structure of coating edges has a considerable impact on an increase in water absorption for the coating in the case of the one-component coating with the polystyrene filler. In the case of the two-component coating, which also has a polystyrene filler (sample 1) the water absorption of the samples with protected cut edges in water at pH 4 is lower compared to the samples of the coating without such protection and exposed to the same environment (3.34% and 4.8% respectively). However, this is only a 30% reduction compared to the reduction for the one-component coating, which was 65% of the maximum value. What is more, the same filler type has a very negative effect on the increase in the water absorption of the two-component product (sample 1) exposed to water with different pH values compared to the values obtained for the one-component product (sample 2). Therefore, it can be presumed that-when it comes to reducing the water absorption of the coating-surrounding the grains of polystyrene filler with bituminous mass to which a powder component is added during the manufacturing process is more effective than the use of the same filler in the two-component product if the powder component is mixed with polymer modified bituminous mass during application on the substrate.

In general, differences in the water absorption of two-component products exposed to demineralized water with a pH of 7.0 and tap water with a pH of 7.5 range from 2 to almost 4%, with smaller differences being observed for the product with the polystyrene filler. These differences narrow when acidic water with a pH of 4 is used, in the case of which the water absorption values for the samples without additional cut edge protection were similar to the values obtained for water with a pH of 7.0. It is evident that the water absorption of two-component polymer modified bituminous thick coatings clearly decreases in slightly basic aqueous solutions, i.e., with a pH of 7.5. Water absorption then increases irregularly even for a small pH decrease (by 0.5), i.e., to a pH of 7.0 until a pH of 4 is reached, at which water absorption remains at the same level. The protection of cut edges in the samples contributes to a considerable decrease in the water absorption of the analysed coatings compared to the results obtained for the samples without such cut edge protection. However, the water absorption value is still higher when exposed to water with a pH of 4 compared to exposure to tap water with a pH of 7.5, especially for two-component products with the polystyrene filler. Both water absorption values, i.e., after soaking in water with a pH of 7.5 and water with a pH of 4, are almost identical (3.45% and 3.34% respectively) only for the two-component product with a polystyrene filler containing 1 mm grains. It should be noted that the pH of 4 is used to assess the effect of a soil-water environment on below ground structures of buildings [37]; therefore, water absorption values of coatings for waterproofing used for such structures may be considered reliable for the assessment of the products in question due to their exposure to water with a pH of 4.

**Figure 4:**
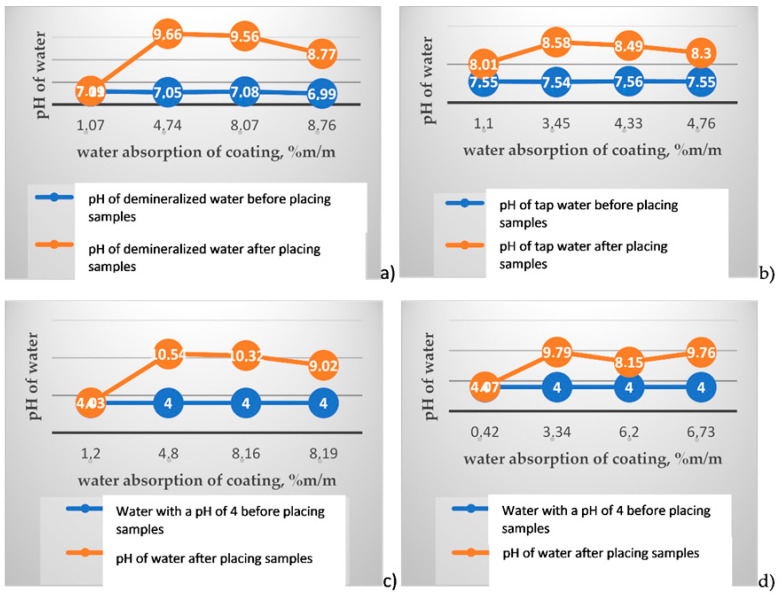
Comparison of water pH values after the test with water pH values before placing the samples in water. (**a**) for demineralized water, (**b**) for tap water, (**c**) for water with a pH of 4 and with the samples whose cut edges were not protected, (**d**) for water with a pH of 4 and with the samples whose cut edges were protected.

After soaking samples of polymer modified bituminous thick coatings for 24 h, a noticeable change in the pH of water can be seen following the removal of the samples from water, which is shown in Figure 4.

**Figure 5:**
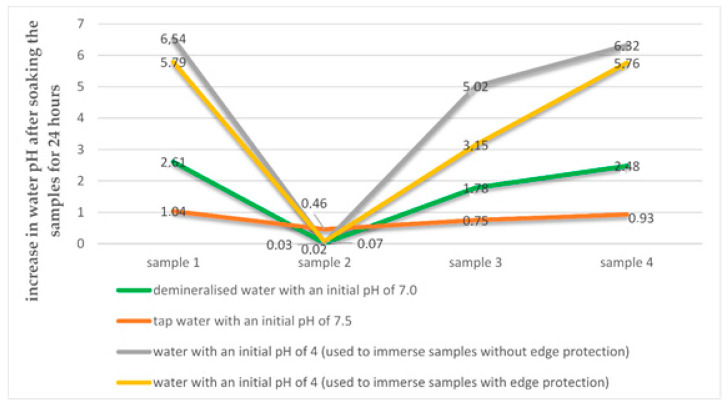
Trends of water pH change during 24 h soaking of polymer modified bituminous thick coatings.

Unfortunately, there is no clear correlation between the increase in coating water absorption and the increase in the pH of the liquid medium in which the sample was tested. For the two-component products, the increase in demineralized water pH after soaking the samples for 24 h ranges from 1.78 to 2.61, meaning that the water is basic after the test. However, the pH value for the one-component product practically does not differ. For soaking the samples in tap water with a pH of 7.5, the change in the water pH after the test is considerably lower and ranges from 0.75 to 1.04 for the two-component products and is equal to 0.46 for the one-component product (see Figure 5). The highest changes in the water pH were observed for polymer modified bituminous thick coatings made of the two-component products tested in water with a pH of 4 and when the cut edges of the samples were additionally protected with wax as well as for the case where such cut edge protection was absent.

The observed phenomenon of water leaching of chemical compounds from polymer modified bituminous thick coatings is consistent with conclusions drawn by Anya Vollpracht et al. [31], formulated by studying these coatings under field conditions, on existing structures. While this study found only small quantities of aromatic hydrocarbon compounds in leachates, the phenol index was above the detection threshold. Admittedly, they did not find measurable quantities of phenol during laboratory leaching tests of the same products with deionized water conducted in parallel. Leaching rate was calculated taking into account: leachate concentration, volume of water, surface area of the coating and length of time of the test. For this reason, Anya Vollpracht et al. [31] assumed that the appearance of phenol in leachates could have been caused by factors related to the use of the studied structures other than leaching from waterproofing layers. Since a change in water pH indicates leaching of alkaline molecules from the coatings, it was additionally checked whether this phenomenon could be observed on the water surface visually assessed, as a change in its color. However, in all tested cases, no change in the color of water used for testing was observed.

**Figure 6:**
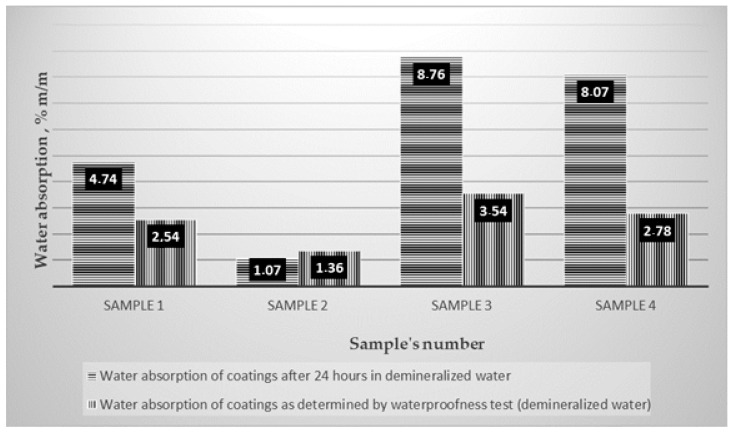
Comparison of water absorption results for coatings obtained using different test methods.

Due to noted increase in water absorption of polymer modified bituminous thick coatings when soaked in demineralized water, the next stage of the study attempted to assess what subsequently happens to water absorbed by the coating. To focus on functionality, assessment concentrated on two probable cases, i.e., further transfer of water collected within the coating to the substrate on which the coating is laid or confinement of water within the coating structure. Form a functional point of view, such assessment is inextricably linked to usefulness of waterproofing coatings. To answer this question, waterproofness of coatings placed on a concrete substrate was tested, with simultaneous assessment of water absorption of these coatings, but this time as a result of exposure to demineralized water with a pH of 7.0 under increased pressure. Results of these tests are summarized in Table 5. There was no water leakage through the coating at 0.5 MPa after testing in any of the tested cases. Concrete substrates were also dry after the coatings had been removed. Due to high susceptibility of polymer modified bituminous thick coatings to deformations appearing at places where point loads are applied, circular depressions were formed on the top side of the sample in the area where the sealing ring was placed during the waterproofness test (Figure 2). After the waterproofness test, the coatings removed from the concrete substrates were additionally assessed for water absorption, this time stemming from long-term, one-sided exposure to water under pressure. Results of these tests were compared with water absorption values for coatings obtained in a 24 h test conducted on samples without additional protection of the cut edges immersed from all sides in demineralized water. A comparison of obtained results is presented graphically in Figure 6.

One-sided exposure to demineralized water under successively increased pressure over 192 h results in lower water absorption of tested coatings compared to exposure to water over 24 h of samples cut from a larger sheet and placed freely in the demineralized water tank. Only in the case of a coating made of one-component mass with a polystyrene filler, this tendency changes and water under pressure causes higher absorbability than tap water with a pH of 7.5, demineralized water with a pH of 7.0 and water with a pH of 4, acting as a free water table. However, these differences are so insignificant that they fall within the measurement error. Unfortunately, this test variant also does not provide a clear pattern for distribution of water absorption of the tested coatings placed on a concrete substrate and subjected to one-sided exposure to pressurized water. However, on the basis of the above comparison it can be concluded that the assessment of polymer modified bituminous thick coatings absorptivity by the method of complete immersion in demineralized water, recommended, e.g., by the standard [39], does not reliably reflect the actual working conditions of the waterproofing layer and therefore should only be used for comparative purposes. For one-sided long-term exposure to demineralized water, including pressurized water, the obtained values of water absorption of polymer modified bituminous thick coatings are much lower than values obtained for water exposure from all sides of the samples. After all, under conditions of use, these coatings are always exposed to one-sided rinsing by water, which is why the authors believe that the method of assessment of this identifying property for the discussed products should be modified. The proposed modification involves placing a cylinder with a repeatable diameter, e.g., 10 cm, on the cured coating surface, filling it with water at approximately 1 m column height, cover the top of the cylinder to prevent evaporation and determining the amount of water absorbed by the coating per 1 m^2^ after 24 h. The proposed solution also does not reflect all conditions of waterproofing movement, including variable pressure acting on the coatings in underground sections of buildings, but at least it partially eliminates the aforementioned shortcomings of assessment of water absorption of coatings by total immersion. Precise specification of criteria useful for evaluation of results obtained using the proposed method requires further research, which is currently in progress.

## 5. Conclusions

This article presents test results related to a functional property of polymer modified bituminous thick coatings which has a significant impact on the usefulness of waterproofing coatings, i.e., the effect of water absorption of coatings on waterproofness. Based on the obtained results, the following conclusions can be drawn:

➢ Water absorbed by the coatings is retained within the layers and is not transferred to concrete substrates on which they are installed, meaning that they provide proper waterproofing,
➢ Water pH has a significant impact on water absorption of polymer modified bituminous thick coatings. The highest water absorption values are observed in the acidic medium, with water pH of about 4, used to assess the resistance of the construction substance to soil and water conditions [18]. Changing water pH towards an alkaline medium (from 7.0 to 7.5) significantly reduces water absorption of polymer modified bituminous thick coatings.
➢ Products with a polystyrene filler show lower susceptibility to water absorption than products with other filler types, which is especially noticeable in one-component products,
➢ Assessment of water absorption of the described products by total water immersion may be used only for comparative purposes and does not fully reflect actual loads acting on coatings under field conditions. For this reason, the article proposes a modification of this test method using one-sided exposure of specimens to water,
➢ Reaction of leachates formed during total immersion of polymer modified bituminous thick coating samples in an aqueous solution with different initial pH values, i.e., 4.0, 7.0 and 7.5, changes significantly towards alkaline, which may indicate a leaching of the coatings which contributes to contamination of groundwater.

## Figures and Tables

**Table 1:** Additional basic properties of the tested products.

Test Sample Number	Type of Product	Mixture/ Component *^)^ Density (g/cm^3^)	pH of a Liquid Component	Mixing Ratio (by Weight)/ Bitumen Emulsion:Powder Component	Average Coating Thickness (mm)
1	two-component polymer modified bituminous coating	1.0	8.64	5:1	4.5
2	one-component polymer modified bituminous coating	0.75	7.50	-	4.6
3	two-component polymer modified bituminous coating	1.07	9.62	3:1	3.6
4	two-component polymer modified bituminous coating	1.15	9.52	3:1	3.7

* Applies to 1-component product.

**Table 2:** Results of demineralized water absorption test of polymer modified bituminous thick coatings.

Test Sample Number	Water Absorption When Using Demineralized Water, % m/m/Coefficient of Variation, %	Water pH	
		Before the Test	After the Test
1	4.74/8.09	7.05	9.66
2	1.07/5.20	7.09	7.11
3	8.76/2.56	6.99	8.77
4	8.07/2.88	7.08	9.56

**Table 3:** Results of tap water absorption test of polymer modified bituminous thick coatings.

Test Sample Number	Water Absorption When Using Tap Water, % m/m/Coefficient of Variation, %	Water pH	
		Before the Test	After the Test
1	3.45/5.57	7.54	8.58
2	1.10/8.32	7.55	8.01
3	4.76/5.58	7.55	8.30
4	4.33/2.76	7.56	8.49

**Table 4:** Summary of results of water with a pH of 4 absorption test of polymer modified bituminous thick coatings.

Test Sample Number	Water with a pH of 4 Absorption % m/m/Coefficient of Variation, %		pH of Water after the Test	
	Samples without Protected Edges	Samples with Wax Protected Edges	Samples without Protected Edges	Samples with Wax Protected Edges
1	4.80/23.11	3.34/10.59	10.54	9.79
2	1.20/13.56	0.42/12.55	4.03	4.07
3	8.19/7.07	6.20/4.92	9.02	8.15
4	8.16/10.04	6.73/16.21	10.32	9.76

**Table 5:** Results of the test of water absorption of coatings and the assessment of their moisture level.

Test Sample Number	Waterproofness, No Leakage at Pressure, MPa	The Concrete Substrate under the Coating after the Test	Water Absorption of Coatings after Waterproofness Test, % m/m/Variation Coefficient, %
1	0.5	No moisture	2.54/4.17
2	0.5	No moisture	1.36/9.71
3	0.5	No moisture	3.54/2.53
4	0.5	No moisture	2.78/3.69

## Data Availability

The data presented in this study are available on request from the corresponding author.
